# Commentary: Bridging the nutritional care gap: nurse-led education for potassium control in hemodialysis patients

**DOI:** 10.3389/fnut.2026.1862782

**Published:** 2026-06-10

**Authors:** Ying Dai, Yinqin Zhong, Fei Yu, Meixia Ye

**Affiliations:** Shenzhen Hospital (Futian) of Guangzhou University of Chinese Medicine, Shenzhen, Guangdong, China

**Keywords:** commentary, hemodialysis, nurse-led education, potassium management, resource-limited settings

## Introduction

A persistent chasm exists between ideal, guideline-driven care and the stark realities of practice in resource-constrained settings. This gap represents one of the most pressing challenges in global nephrology. The study by Ouirdani et al. shines a necessary and pragmatic light on this very issue, investigating a nurse-led educational intervention for potassium management in Moroccan hemodialysis centers operating without dietitians. While the pilot nature of the work precludes definitive conclusions, it courageously addresses a critical question: can task-shifting and simplified educational strategies mitigate the adverse outcomes stemming from specialist shortages? This commentary aims to contextualize the study's findings within the broader evidence landscape, critically appraise its methodological framework, and offer constructive guidance for future research seeking to bridge this pervasive care gap.

## Feasibility and contextual relevance

The core strength of this research lies in its authentic engagement with a real-world problem. The authors correctly identify the severe shortage of dietitians in the Moroccan public health system as a fundamental barrier to implementing standard nutritional care. In this context, exploring the feasibility of nurse-led education is not merely an academic exercise but a vital step toward pragmatic solutions. The reported significant reduction in serum potassium levels (*p* = 0.002) and the increase in the proportion of patients within the target range (from 36.7% to 46.7%) are encouraging preliminary signals. These findings align with a growing body of evidence supporting the role of structured, nurse-delivered education in improving specific patient outcomes in chronic disease management (1). The intervention was designed to utilize visual aids and was delivered in the local dialect. This design demonstrates an adaptation appropriate for the study population's cultural context and literacy level, given that 70% had no formal education. This pragmatic approach is commendable and essential for any intervention intended for scale in similar settings.

## Methodological considerations and unexplored pathways

However, the study's quasi-experimental, single-arm, pre-post-design inherently limits the strength of inference. The absence of a control group makes it difficult to attribute the observed potassium improvement solely to the intervention, as secular trends or other concurrent care changes cannot be ruled out. Furthermore, the lack of significant change in interdialytic weight gain (IDWG) and quality of life (QoL) warrants deeper scrutiny. While the authors suggest that more intensive or individualized interventions may be needed, alternative explanations rooted in the study's design should be considered. Variability in how the intervention was delivered (e.g., face-to-face in one center vs. a hybrid model in others) may have introduced performance bias. This inconsistency could have diluted the intervention's effect on complex behaviors, such as fluid adherence. The focus on potassium, while important, may have been too narrow. A recent systematic review and meta-analysis on nutritional education in hemodialysis patients concluded that while interventions often favor the experimental group, significant improvements in comprehensive biochemical panels are not consistently achieved, and success is more closely tied to collaborative care models and active patient engagement in meal preparation (2). The focused single-nutrient approach taken in the present study, therefore, represents a significant limitation. It missed the opportunity to assess the intervention's broader impact on nutritional management, which is critical in chronic kidney disease-mineral and bone disorder (CKD-MBD). This aligns with the findings of a systematic review (2), which suggests that successful nutritional education in hemodialysis (HD) patients is more consistently associated with collaborative, multi-component care models rather than single-nutrient approaches.

## Toward a more holistic and sustainable model

**Figure 1:**
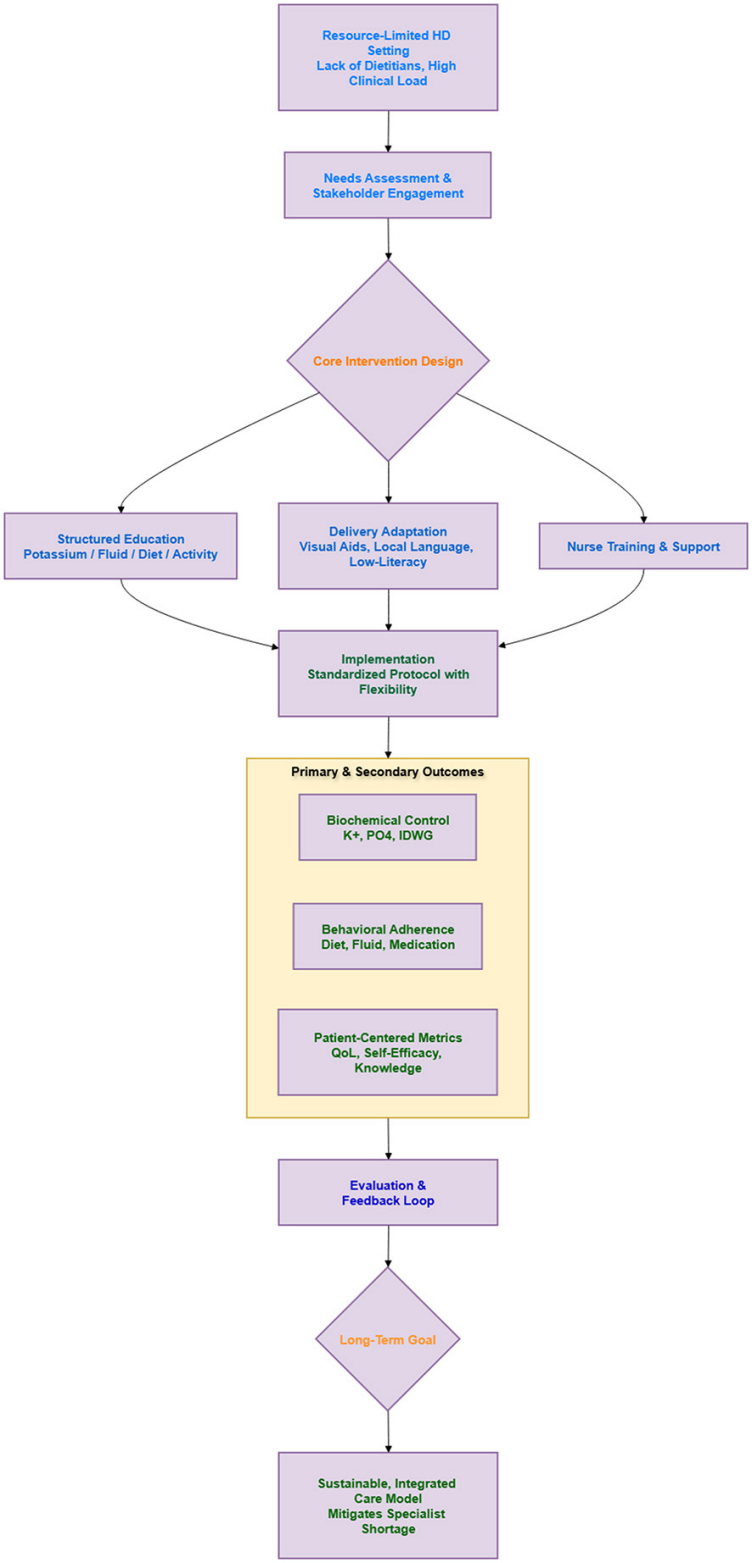
A proposed conceptual framework for developing and evaluating multifaceted nutritional interventions in resource-limited hemodialysis settings. The framework outlines a cyclical process, beginning with a needs assessment in the target setting and progressing through the core design of adapted education, its implementation, and the measurement of multi-dimensional outcomes. The process incorporates an evaluation and feedback loop, with the ultimate goal of establishing a sustainable, integrated care model that mitigates specialist shortages. The color scheme utilizes blue for foundational elements and processes, orange for key decision or design points, and green for targeted outcomes and the long-term goal.

The study rightly concludes that larger, controlled trials are needed. Future research could build on this foundation by adopting cluster-randomized designs to account for center-level effects and including an active control group receiving standard care. The intervention itself could be enhanced by integrating a more holistic nutritional curriculum and employing behavioral change techniques tailored to low-literacy populations, such as teach-back methods or peer support. Given the notably high proportion of participants with no formal education (70%) identified in the study, addressing the challenge of sustaining behavioral change in low-literacy populations is crucial. Future interventions could integrate structured behavioral change techniques (e.g., teach-back, peer support) and leverage technology. For instance, incorporating technology, such as mobile applications for food logging or telenursing for follow-up, has shown promise in improving nutritional markers and self-efficacy in this patient population (3). Crucially, assessing sustainability requires longer follow-up periods to determine if knowledge translates into lasting behavioral change. The conceptual pathway below (Figure 1) outlines a potential framework for developing and evaluating such multifaceted interventions in resource-limited environments.

## Discussion

In summary, the pilot study by Ouirdani et al. provides a valuable proof-of-concept for task-shifting nutritional education to nurses in resource-scarce settings. However, the quasi-experimental design and narrow biochemical focus highlight the inherent limitations of such preliminary work and underscore the need for more rigorous investigation. The true contribution of this research may lie less in its specific findings and more in the potential direction it identifies: to bridge the pervasive nutritional care gap, future initiatives could ideally progress in two key dimensions. First, methodologically, from demonstrating feasibility to establishing efficacy through robust, controlled trials. Second, and more importantly, conceptually—from isolated, single-nutrient education toward integrated, theory-informed behavioral interventions that address the holistic and sustained dietary management required in CKD. This two-pronged approach is essential for developing more robust models needed to translate pragmatic adaptations into measurable, long-term benefits.
